# Assessing Risk Among Correctional Community Probation Populations: Predicting Reoffense With Mobile Neurocognitive Assessment Software

**DOI:** 10.3389/fpsyg.2019.02926

**Published:** 2020-01-24

**Authors:** Gabe Haarsma, Sasha Davenport, Devonte C. White, Pablo A. Ormachea, Erin Sheena, David M. Eagleman

**Affiliations:** ^1^The Center for Science and Law, Houston, TX, United States; ^2^Administration of Justice Department, Texas Southern University, Houston, TX, United States; ^3^Department of Psychiatry and Behavioral Sciences, Stanford University School of Medicine, Stanford, CA, United States

**Keywords:** risk assessment, machine learning, neurolaw, predictive validity, neurocognitive

## Abstract

We seek to address current limitations of forensic risk assessments by introducing the first mobile, self-scoring, risk assessment software that relies on neurocognitive testing to predict reoffense. This assessment, run entirely on a tablet, measures decision-making via a suite of neurocognitive tests in less than 30 minutes. The software measures several cognitive and decision-making traits of the user, including impulsivity, empathy, aggression, and several other traits linked to reoffending. Our analysis measured whether this assessment successfully predicted recidivism by testing probationers in a large urban city (Houston, TX, United States) from 2017 to 2019. To determine predictive validity, we used machine learning to yield cross-validated receiver–operator characteristics. Results gave a recidivism prediction value of 0.70, making it comparable to commonly used risk assessments. This novel approach diverges from traditional self-reporting, interview-based, and criminal-records-based approaches, and can also add a protective layer against bias, while strengthening model accuracy in predicting reoffense. In addition, subjectivity is eliminated and time-consuming administrative efforts are reduced. With continued data collection, this approach opens the possibility of identifying different levels of recidivism risk, by crime type, for any age, or gender, and seeks to steer individuals appropriately toward rehabilitative programs. Suggestions for future research directions are provided.

## Introduction

The Bureau of Justice Statistics estimates that 12–13 million people are processed annually through jail facilities nationwide – and that 68% of released felony-level prisoners are rearrested within 3 years, 79% within 6 years, and 83% within 9 years (Alper et al., 2018). The criminal justice system has long seen the value in determining the best course of treatment, sentencing, or release of an offender by administering tests or reviewing records to roughly classifying individuals in terms of future risk of rearrest (Berk, 2017). However, concerns about the fairness and accuracy of risk assessments (Eckhouse et al., 2019) have increased the stakes that prosecutors and judges face when making risk-based determinations. The development of actuarial risk assessments sought to address inadequacies and provide statistical soundness to the approach of using only clinical judgment and criminal history (Sreenivasan et al., 2000). It follows that a statistically sophisticated tool which could uncover predictive traits and dynamic factors at the individual level, while filtering out unfair biases, used in conjunction with clinical judgment, would be beneficial to persons in the system and society.

Forecasting an individual’s likelihood of future criminality has been part of the criminal justice system “since judges have been judging” (Gottfredson, 1987; Berk and Hyatt, 2015). Methodologies have expanded the scope of assessing risk of reoffending, and over the past 40 years courts have become significantly more advanced in attempting to divide high- from low-risk offenders. The predicted level of risk can be used to determine pretrial release, steer bail amount (Desmarais and Lowder, 2019), length of sentence, or probation status, and it can also shed light on rehabilitation strategies. Having an idea of the risk someone poses to the public can allow courts to more optimally produce sentences to balance freedoms against societal protection.

Individuals are quite different in their predispositions (Eagleman, 2011), and because lives are complex, and crime is contextual, there will never be a test that accurately predicts the future (such as the “pre-cogs” in the movie *Minority Report*); nonetheless, risk assessments have been shown to perform at a higher rate of accuracy than subjectivity of psychiatrists and parole boards (Grove et al., 2000; Ægisdóttir et al., 2006; Spohn, 2008; Dressel and Farid, 2018). There are over 60 risk assessments used to specifically measure recidivism in the United States (Barry-Jester et al., 2015; Casselman and Goldstein, 2015). In this paper, we present 17 of them (Table 1) to establish a landscape of the most widely used tests.

**TABLE 1:** Commonly used risk assessments, their stated purpose, and their median area under the curve (AUC) (Singh et al., 2011; Desmarais et al., 2016).

**Risk assessment**	**AUC**	**Purpose**
COMPAS	0.67	General and violent recidivism, pretrial misconduct
IRAS	0.63	General recidivism
LSI-R	0.64	General recidivism
ORAS	0.66	General recidivism
PCRA	0.71	Post-conviction reoffense, under supervision
PSA	0.66	Pretrial risk assessment
RMS	0.67	General recidivism
SARA	0.70	Domestic violence
SAVRY	0.71	Violent risk in youth
SORAG	0.75	Sex offender
SPIn-W	0.73	Gender-responsive (for women)
Static-99	0.70	Sex offenders, pre-release
STRONG	0.74	General recidivism
SVR-20	0.78	Sexual violence
TRAS	0.67	General recidivism
VRAG	0.74	Violent risk
WRN	0.67	General recidivism

*COMPAS, Correctional Offender Management Profile for Alternative Sanction; IRAS, Indiana Risk Assessment Survey; LSI-R:SV, Level of Service Inventory; ORAS, Ohio Risk Assessment Survey; PCRA, Federal Post Conviction Risk Assessment; PSA, Public Safety Assessment; RMS, Risk Management System; SARA, Spousal Assault Risk Assessment; SAVRY, Structured Assessment of Violence Risk in Youth; SORAG, Sex Offender Risk Appraisal Guide; SPIn-W, Service Planning Instrument–Women; Static-99; STRONG, Static Risk and Offender Needs Guide; SVR-20, Sexual Violence RIsk-20; TRAS, Texas Risk Assessment Survey; VRAG, Violence Risk Appraisal Guide; WRN, Wisconsin Risk and Needs.*

Most risk assessments ask questions that measure factors that are classified as static or dynamic (Austin, 2004; Desmarias, 2013). Early risk assessments that used prior arrest records or interview-based assessments predominantly focused on static factors – that is, variables that cannot be changed (race, place of birth, or arrest record). Some such static factors are used regularly to inform rehabilitation tracks, such as gender, age, and crime type. On the other hand, some researchers are concerned that static factors yield a risk score that is too restrictive, because such factors do not allow for the possibility that an individual can change.

By contrast, traits that can change over time (called dynamic factors) offer more indication of an offender’s current and future behavior (Andrews and Dowden, 2007; Ward and Fortune, 2016). These include factors such as education, employment, marital status, and cognitive traits. Dynamic risk factors can be mitigated with intervention strategies (Bonta and Andrews, 2007). Some researchers have developed assessment tools that combine static and dynamic factors to estimate the likelihood of reoffending and offer appropriate recommendations. This provides correctional professionals with a baseline for determining risk while allowing for change over time as well.

The most commonly used actuarial risk assessments ask similar questions about the individual, and share similar predictive strength as measured by the receiver operating characteristic curve (ROC curve), and the area under the curve (AUC), which ranges from 0.5 (no predictability) to 1 (perfect predictability). This value serves as an evaluation metric of how good the models are at distinguishing between two classes. Models are built to make probability predictions about each participant’s chance of falling into two classes: those who will recidivate, and those who will not. The higher the ROC AUC is, the better it is at classifying between the two groups.

The best assessments range in AUC values from the mid-0.6s to mid-0.7s. Some risk assessments measure risk for specific crimes, or populations, such as STATIC-99 and SAVRY (Structured Assessment of Violence Risk in Youth), which concentrate specifically on sex crimes and risk of violence for youth, respectively. However, note that some studies indicate that having a high predictive value for some measures (such as sex crimes) correlates with low predictive value for other serious crimes (Langton et al., 2007). Thus, these more specific risk assessments are narrower in their predictive capacities.

While current risk assessments have been successful, they also have limitations. First, the information used to score the assessments is generally gathered from a single criminal offense level and may not be flexible enough to apply to another. For example, if a risk assessment is validated to score well at the felony level, it is not guaranteed to be accurate at the misdemeanor level (Pope-Sussman and Turner, 2015). Second, in the absence of expensive, ongoing training, the variance between rater scores can be a concern (Lowenkamp et al., 2004; Duwe, 2017). Third, actuarial risk assessments can be time consuming, affecting key stakeholders such as administration, practitioners, and test takers (Desmarias, 2013). Fourth, there may be problems in taking an assessment that was validated at one point of the criminal justice pipeline and using it in a different application for which it may not be as fair and accurate, e.g., pre-trial vs. recidivism (Dressel and Farid, 2018). Further, recent studies and lawsuits have highlighted the possibility of racial bias when an assessment relies on subjectivity of the interviewer, as well as using static and dynamic factors that correlate with race (e.g., education and previous criminal history) (Harcourt, 2015; Dressel and Farid, 2018). Although race is not included in risk assessments, many factors included in risk assessments correlate heavily with race. In a 2014 speech to the National Association of Criminal Defense Lawyers, former Attorney General Eric Holder warned that sentencing decisions based on “immutable characteristics may exacerbate unwarranted and unjust disparities that are already far too common in our criminal justice system and in our society” (Holder, 2014). Recent court rulings have further highlighted the unfairness of the use of proprietary scoring algorithms that do not allow one to see how the score was calculated, and thus assess its accuracy or contest the score (State v. Loomis, 2016; Kehl et al., 2017).

**FIGURE 1:**
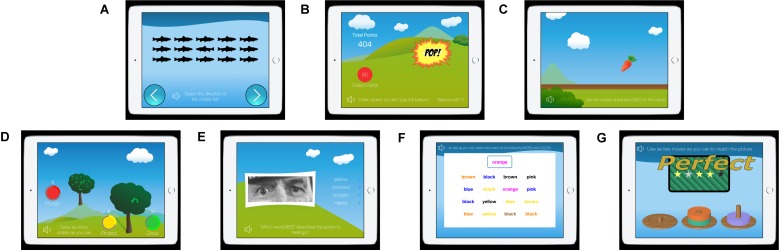
Screenshots of the NeuroCognitive Risk Assessment (NCRA): **(A)** the Eriksen Flanker, **(B)** Balloon Analog Risk Task, **(C)** Go/No-Go, **(D)** Point-Subtraction Aggression Paradigm, **(E)** Reading the Mind Through the Eyes, **(F)** Emotional Stroop, and **(G)** Tower of London (Ormachea et al., 2017).

To address these limitations, we have developed an innovative assessment tool for predicting reoffense using rapid, interactive tests based on standard neuropsychological tests (Ormachea et al., 2017; Figure 1). The NeuroCognitive Risk Assessment (NCRA) measures key criminogenic factors (attentiveness, aggression, risk seeking, empathy, future planning, emotional processing, and impulsivity), all of which have been identified in the literature as cognitive traits linked to reoffending. We then used machine learning models to quantify an individual’s risk for re-offense, which yields findings significantly better than using general linear modeling alone.

There are several benefits to the NCRA. The test is self-administered on a mobile device (such as an iPad), and test administrators require no training to supervise individuals taking the test. The interactive battery is “gamified”, making the test interactive and engaging compared to traditional questionnaires (Table 1). Administrators are not required to have extensive training, or a professional degree to interpret the results, and they do not need to directly administer the assessment to participants individually while they are taking the test, thus allowing it to be taken in a group setting. The test is self-administered by the participant, and the visual and audible instructions, along with the practice rounds, make the battery easy to understand, even among low literacy populations (the current version requires only a 4th grade reading level). Further, the minimal text in the games is easily translated into different languages, allowing testing in different tongues and locations. Collectively, these qualities make adoption of the NCRA more accessible, scalable, affordable, and less time/resource consuming than traditional assessments.

Analysis of NCRA scores is based on machine learning and therefore can be grouped with the most current risk assessment tools as an actuarial method (Berk and Hyatt, 2015). It can inform case management by allowing the ongoing tracking of decision-making traits while a person participates in programs, which can be useful in case planning or identifying levels of service, needs, or risk management. For example, knowing an offender’s aggression and impulsivity profile could assist case workers in monitoring progress through treatment programs and target more effective behavioral therapies. Rather than using static factors such as criminal history or demographics – which often come under scrutiny for potential bias – the NCRA measures dynamic cognitive factors in decision-making such as risk taking, aggression, empathy, impulsivity, and attention – all of which are dynamic traits that can be improved.

Predictive validity is a spectrum, in which scores estimate the likelihood of a person recidivating. Assessed over a population, an assessment can be measured by its AUC value, where 0.5 depicts no discriminative ability, 1.0 represents perfect prediction, and most good risk assessments have an AUC of around 0.70 (Howard, 2016). The most commonly used risk assessments have virtually the same predictive validities, so there is little in terms of a hierarchy of effectiveness between these different risk assessments (Campbell et al., 2009; Yang et al., 2010; Table 1). This is because many of the most commonly used risk assessments use essentially the same factors, self-reporting and weighted questions about the individual past and current position, and only differ in the way they analyze those factors (Monahan and Skeem, 2014), typically with proprietary algorithms that cannot be studied. Because they are using the similar risk factors and have the similar AUC predictive validity scores, the risk assessments might have reached a “glass ceiling” that cannot be broken (Monahan and Skeem, 2014). In other words, to attain a more accurate risk assessment, the input predictive factors must differ from using *only* interviews, self-reporting questionnaires, or records-based assessments, as well as having a defined outcome (Fazel et al., 2012). Utilizing cognitive traits of the NCRA offers inputs that allow new predictive features to emerge, ones that can also inform rehabilitation needs. It bears re-emphasizing that it is impossible for risk assessment to predict whether someone will recidivate with 100% accuracy, because people are unpredictable, life is complex, and crime can be contextual. Nonetheless, in the same way that life insurance companies improve their returns by building actuarial tables to assess whether a customer is high-, medium-, or low-risk, perfect predictability is not necessary to improve sentencing and rehabilitation decisions in the criminal justice system.

In this study, we use machine learning to examine the predictive validity of the NCRA in a forensic community corrections population. The literature in computer science has demonstrated that machine learning statistics can forecast more accurately than previous approaches based on regression analysis (Trevor et al., 2009; Berk and Hyatt, 2015; Duwe and Rocque, 2017). These machine learning techniques differ from the typical statistical analysis used in conventional risk assessments that often have anticipated and weighted relationships between crime and recidivism that are then built into the model. By contrast, machine learning models finds relationships within the data that are not prescripted, and may not be obvious, like interactions or non-linear relationships, but nonetheless increase predictive validity and give a near-optimal prediction of recidivism (Berk and Hyatt, 2015).

Moreover, the NCRA eliminates factors that discriminate against individuals based on race and socioeconomic status, because the assessment does not need to compute data that are linked to these characteristics. Rather, the NCRA uses only neurocognitive measures (which assess attributes linked with criminality and reoffense) that can be modified and improved.

## Materials and Methods

### Study Design

Informed consent was obtained from 730 probationers who volunteered to participate in the study. Participants self-administered the tablet-based test in about 30 min. All participants were provided headphones for the auditory and visual test instructions. They reviewed instructions and played brief unscored practice tests prior to each assessment to ensure they understood each test’s rules. Participants were not offered any reward or compensation for participating in the study, and were debriefed afterward.

Once consent was obtained, the mobile device was handed to the participant. The battery began with a short customizable questionnaire for participants to enter demographics or answer questions relevant to the program or facility they were in. Each test began with video-based, audible instructions, using language and text designed for a 4th grade reading level. Non-scored practice rounds were offered after the instructions, which included feedback to ensure the test-taker fully understood the instructions. The NCRA is comprised of seven tests (Figure 1), each of which was selected based on their relationship to reoffense, as detailed in the neurocognitive literature (Ormachea et al., 2017). The tests were then “gamified” (to benefit engagement and attention) and optimized to balance data collection against rapid testing time.

The seven tests, as deployed in conjunction with one another, does not exist anywhere else, so the NCRA is unique, despite growing from tests that have been historically used to analyze neurocognitive behavior. Each test lasts 2–4 min and (depending on the speed of the test taker) the entire battery is administered in about 30 min. After the participant completes each test, scores are automatically calculated by the software. The results are stored with HIPAA-compliant security in the cloud.

To analyze results, NCRA test data were taken for each participant, along with age, gender, and current offense category (Tables 2, 3) participants were charged with at the time of testing. Two publicly available criminal history databases were used to ensure the most accurate information. Information from the state obtained through the county probation department was used to track reoffenses, or any arrest that happened post assessment.

**TABLE 2:** Overview of probationers (age and gender) and recidivism, by current offense category.

**Category**	***N***	**Recidivate (*N*)**	**Recidivate (%)**	**Gender (Male%)**	**Age (Median)**	**Age (SD)**
DWI	182	15	8.2	78.0	31.9	10.7
Drug	187	38	20.3	78.6	28.4	10.0
Non-violent	35	13	37.1	85.7	29.1	9.3
Property	122	23	18.9	59.8	27.7	10.2
Sexual non-violent	8	1	12.5	75.0	29.4	8.9
Sexual violent	8	1	12.5	87.5	38.6	10.1
Violent	188	35	18.6	77.1	27.9	8.6
Total	730	126	17.3	75.3	28.8	10.0

**TABLE 3:** Self-reported race/ethnicity and number of previous arrests.

		**Race/ethnicity (%)**					**Arrests (*N*)**					
**Category**	***N***	**Asian**	**Black**	**Hispanic**	**White**	**Other**	**0**	**1**	**2**	**3**	**4–10**	**11**+
DWI	182	3.3	23.1	41.8	27.5	4.4	10	73	41	27	31	0
Drug	187	3.2	32.1	34.8	22.5	7.5	5	49	40	50	41	2
Non-violent	35	0.0	65.7	28.6	2.9	2.9	2	8	9	9	7	0
Property	122	1.6	44.3	31.1	22.1	0.8	8	46	26	20	22	0
Sexual non-violent	8	0.0	50.0	25.0	25.0	0.0	0	3	2	1	1	1
Sexual violent	8	0.0	25.0	25.0	25.0	25.0	0	5	1	1	1	0
Violent	188	1.1	44.7	32.4	16.5	5.3	11	53	42	48	31	3
Total	730	2.2	36.8	34.8	21.2	4.9	36	237	161	156	134	6

*Note that none of this self-reported information is used in any of the machine learning sections.*

### Participants

The NCRA was self-administered by 730 participants in the Harris County Community Supervision and Corrections Department. We tracked reoffense of participants by utilizing two data sources: the Harris County District Clerk public criminal records database and the Texas Department of Public Safety. The earliest check on rearrest was conducted at 4 months post-assessment, and re-checked regularly up to 2 years post-assessment. Recidivism is defined by any subsequent arrest after the initial arrest (Andrews and Bonta, 1995; Markman et al., 2016). Technical violations of conditions of probation (e.g., failing to update current address or missing an appointment) was not counted, even if the event resulted in adjusted probation terms.

The testing group comprised adult participants recruited from the Harris County Community Services and Corrections Department (CSCD) from 2017 to 2019. Of the participants, 550 (75.3%) were male and 180 (24.7%) were female. Participants had either been assigned to probation through the court from a previous arrest, or were in pre-trial assessments for a recent arrest. Participants were charged with misdemeanors (332) or felonies (398). Descriptive statistics of participants population are provided in Tables 2–4.

**TABLE 4:** Self-reported education and employment.

**Education**	***N***	**(%)**	**Employment**	***N***	**(%)**
Middle or junior high	17	2.3	Not employed	152	20.8
Some high school	143	19.6	Homemaker	14	1.9
High school or GED	323	44.2	Student	47	6.4
Some/in college	154	21.1	Employed	380	52.1
College graduate	36	4.9	Student/employed	12	1.6
Graduate school	7	1.0	Other	125	17.1
Vocational school	50	6.8			

*Note that education and employment information is not used in any of the machine learning sections.*

In 2 years, 126 of the 730 participating probationers (17.3%) recidivated in Harris County (Table 2). This is an underestimation of the actual recidivism of the offenders, as some crime goes undetected (for example, as happens in jurisdictions we do not have access to). Also, Class C misdemeanors (as defined by the Texas State penal code) were left out of this analysis – e.g., crimes that result in no jail time and have fines <$500.

### About the Assessment

Throughout the development of the NCRA, our aim has been to determine how underlying cognitive traits (and specifically, those that have established links to criminal behavior) can be used to harvest insights into recidivism. An appreciation of how these decision-making traits are linked to reoffending can optimize individualized sentencing strategies, and can steer rehabilitative program recommendations toward individualized treatment (Ormachea et al., 2016). We’ve leveraged neuropsychological tests that are sensitive to different cognitive domains, gamified them, and time-optimized them. By running them on a tablet, accuracy and reaction time (down to the millisecond scale) can inform scoring (Zelazo et al., 2014). The following is a brief description of the tests:

The *Eriksen Flanker task* is a focus and attention task that measures executive functioning. A school of fish that is heading left or right is displayed on the screen. The middle fish may point in the same direction (*congruent*) or a different direction than the school (*incongruent*). The object of the game is to press an arrow on the screen indicating the direction that only the middle fish is facing, ignoring all other distracting fish.

The *Balloon Analog Risk task* (*BART*) was chosen to measure risk taking behavior. The object is to inflate the balloon by pressing on it, as much as you dare, earning points as the balloon continues to grow. But beware, the balloon could burst at any time and all points “risked” for that trial will be lost.

The *Go/No-Go* (*GNG*) *task* measures a participant’s ability to inhibit impulsivity. The aim is to touch the screen as fast as possible when a carrot is plucked up from the ground. However, in a fraction of trials, an eggplant pops up instead of a carrot, and in this circumstance the user is meant to inhibit the urge to tap.

The *Point-Subtraction Aggression Paradigm* (*PSAP*) is a test that measures reactive aggression. The aim is to grow dollars on the tree as fast as possible by rapidly tapping the “grow” button. However, a second player (who is actually the computer) is trying to grow money on their tree as well, and will sometimes “steal” dollars from the participant’s tree, resulting in two more choices that appear: the participant can protect the dollars they’ve grown so far, or retaliate and “punish” player 2 by eliminating one of their dollars. Either of those choices requires multiple taps and takes time away from the participants clear instructions, which is to “grow” as much money as they can. The game measures how aggressively a participant is prone to react to a slight.

The *Reading the Mind Through the Eyes* (*RTMTE*) *task* measures social cognition, specifically, empathy, which tends to be deficient in violent offenders (Baron-Cohen et al., 1997; Domes et al., 2013; Seidel et al., 2013). Users are presented with the upper half of a face. They are tasked with selecting a word (out of four words) that best describes the face’s emotional state. Of 30 trials, we track the number that are incorrect.

The *Emotional-Stroop test* detects microsecond apprehensions related to the negatively charged words. Consisting of several levels, the user starts with a traditional Stroop test involving words and colors (neutral, congruent, incongruent). The final levels introduce series of neutral words, positive words, and negatively charged words (related to drug use), in the same format. The tests pick up on delays of negative words that require more emotional processing time and indicate a relationship.

The *Tower of London* (*TOL*) is a shape- and color-matching game that tests the ability to simulate future consequences and plan ahead. The user is shown three pegs that up to three colored discs are stacked on. The task is to make the fewest moves possible to match the pattern of disks shown.

After the participants completed the NCRA, we regularly queried databases for evidence of recidivism, as well as the crime category and level (misdemeanor vs. felony). For this, we used the Harris County District Clerk public criminal records database and the Texas Department of Public Safety criminal history search. The former was automatically queried monthly, and provides detailed records about offenses from Harris County. This was augmented with additional arrest and court data from the Texas Department of Public Safety, which was queried semi-annually for offenses committed outside Harris County.

### Features Used and Feature Sets

Each neurocognitive test generates raw unstructured data, called features. These typically involve the individual trial number, specific information about the trial, millisecond resolution timing when a button was pressed, and whether the individual answer or action was correct or incorrect. From the raw unstructured data, machine learning features were developed for each test. These are generally a set of summary statistics for the test (Table 5). Beyond these features, participants’ age and gender demographics, as well as the current offense category were used. We never included other variables, such as race/ethnicity, education, or employment.

**TABLE 5:** Definitions of the machine learning features used in each NCRA test.

**Feature**	**Description**
**Eriksen Flanker**	
Time median	Median response time
Time standard deviation	Standard deviation response time
Exec effect	Median congruent trials – median incongruent trials
Frac correct	Percent of correct trials
NIH score	National Institute of Health Flanker score
**Balloon analog risk task**	
Pop	Number of popped balloons
Time collected (*)	Total time/points collected from unpopped balloons
Pressed time median	Median time/points collected
Pressed count median	Median number of balloon inflate presses
Duration time median	Median time/duration of inflate presses
**Go/no-go**	
Correct go	Correct number of Go’s (carrot)
Correct no go	Correct number of No-Go’s (eggplant)
Time correct go	Mean response time of correct Go’s
**Point-subtraction aggression paradigm**	
Grow (*)	Number of individual grow taps/50
Protect ratio	Protect taps/all taps
Punish ratio (*)	Punish taps/all taps
**Reading the mind through the eyes**	
Correct (*)	Number of correct trials
Time median (*)	Median response time
Time standard deviation	Standard deviation response time
Dict lookup	Number of trials any trial word is looked up
**Emotional Stroop**	
Test correct	Test round with feedback number of correct trials
Test time (*)	Test round with feedback mean response time
Black correct	Std Stroop color words in black number of correct trials
Black time (*)	Std Stroop color words in black mean response time
Con color correct	Std Stroop color words congruent color number of correct trials
Con color time	Std Stroop color words congruent color mean response time
Incon color correct	Std Stroop color words incongruent color number of correct trials
Incon color time (*)	Std Stroop color words incongruent color mean response time
Neutral correct	Neutral words number of correct trials
Neutral time	Neutral words mean response time
Pos Neg correct	Positive and negative words number of correct trials
Pos Neg time (*)	Positive and negative words mean response time
**Tower of London**	
Solved	Number of trials solved
Aborted (*)	Number of trials aborted (giving up)
All moves	Number of total moves
Dup moves (*)	Number of duplicated moves
Extra moves	Number of extra moves to solve
Illegal moves (*)	Number of illegal moves
Mean time	Mean trial time
Solved mean time	Mean trial time for solved trials
Solved median time	Median trial time for solved trials
First move time	Mean time waited before moving a disk in a trial
First move frac (*)	Mean fraction of time waited before moving a disk in a trial
Final time	Time the last disk was moved
Test moves	Number moves in the test round
Test time	Time spend in the test round
Test solved	Was the test round solved
Disk speed	Mean time between start and stop of moving a disk

*Asterisks signify the most predictive features.*

By analyzing the NCRA feature data alone and then combining the NCRA with basic information about the participant (age, gender, and current offense category), we filtered for the most predictive NRCA features and introduced four different feature sets (Table 6).

**TABLE 6:** Feature sets defined, as used in machine learning modeling analysis.

**Feature set**	**Description**
Full NCRA	NCRA test data, without any other information
RFE NCRA	Recursive feature elimination producing the top 13 most predictive features of the NCRA, with no other information
Full NCRA + Demographics	NCRA test data combined with demographics (age, gender, and the current crime category at the time of testing)
RFE NCRA + Demographics	Recursive feature elimination producing the top 13 most predictive features of the NCRA combined with demographics

### Machine Learning and Predictive Validity

Traditionally, the most suitable method for estimating and predicting the probability of an event at a single point in time is a standard linear logistic regression (generalized linear models or glm). To take advantage of newly developed advances in data science, we applied machine learning methods to optimize the feature selection and address possible non-linearities in the data. Machine learning has a number of benefits over traditional statistical methods, including efficient handling of noisy data, non-linearities, and numerous predictors, as well as being able to automatically mine and estimate complex interactions (Tollenaar and van der Heijden, 2013). To build on this, we report the findings of both the generalized linear regression along with machine learning packages (Table 7).

**TABLE 7:** Machine learning models used with corresponding R statistical analysis package.

**Label**	**Method**	**R package**
GLM	Generalized linear models	stats version 3.6.1
LDA	Linear discriminant analysis	MASS version 7.3-51.4
k-NN	k-Nearest neighbors	class version 7.3-15
SVM	Support vector machines (polynomial)	kernlab version 0.9-27
GMB	Generalized boosted modeling	gbm version 2.1.5
RF	Random forest	ranger version 0.11.2
Glmnet	GLM with ridge and lasso regularization	glmnet version 2.0-18

Predictive validity describes the degree to which a score predicts a criterion measure – in our case, recidivism. To assess the predictive validity of the machine learning methods chosen, the focus of this paper is on the ROC curve, which plots the true positive rate (sensitivity) against the false positive rate (1-specificity) for every possible cut-off threshold. An ROC curve captures the predictive ability of a binary classifier system (in this case, *recidivates* or *does not recidivate*). When using such a plot, one traditionally measures the AUC, which gives the probability that any given classifier will rank a randomly chosen positive instance higher than a randomly chosen negative one (Hanley and Mcneil, 1982). A perfect model which completely separates the two classes would have 100% sensitivity and specificity, which will result in an AUC of 1. In contrast, a completely ineffective model would result in a ROC curve that closely follows the diagonal line and would have an area under the ROC curve of approximately 0.5 (Kuhn and Johnson, 2013). We measure the NCRA via the ROC AUC, as this is the most widely used method in measuring predictive validity in risk assessments (Rice and Harris, 2005; Singh et al., 2013). The higher the AUC, the more accurate the model is in predicting (Cortes and Mohri, 2003; Clémençon and Vayatis, 2010).

### The Challenges of Small and Unbalanced Data Sets in Machine Learning

Large datasets are better for machine learning, so additional care has to be taken when working with smaller sets. Despite our dataset being sizable when compared to traditional risk assessment validation studies (Singh et al., 2011), the data are used for both the development/training of the model and the validation of it. The first concern is the overfitting of data, which can lead to low errors in training, but high variance when using the model on a hold-out validation test. This error gets amplified when using high dimensional, noisy data (such as human behavior) that drowns out the nuances and leads to poorly generalizable results.

Many of the machine learning algorithms and models that are used require one or more tuning parameters to be set that have a large effect on their performance. In each type of model we must select the model that performs best in a range of tuning parameters. To select the best model over its range within the estimation set, we use five times repeated 10-fold cross-validation. The repeated cross validation (RCV) will select the optimal set of tuning parameters for a given machine learning algorithm. After optimal model parameters are established, 80% of the data is used to construct a final model. The other 20% is used as a hold-out set which is used to evaluate the performance of the final candidate model.

Both the model training and hold-out validation set are randomly split while balancing the recidivism class. A single training and validation run has significant variability in predictive validity from the random split. To address this, the splitting training and validation are repeated up to 100 times with different random seeds for the split. This will create a distribution from which to get an average, the results of the repeated samples represent the validity of the model’s predictions, which leads to a ROC, which is then used to create an AUC.

A dataset is said to be unbalanced when the class of interest (minority class) is much rarer than normal behavior (majority class). The overall recidivism rate is 17.3% (our minority class), which creates a mild imbalance in the data. This kind of mild imbalance is not a problem for most machine learning algorithms. However, when combined with the small overall data set, it is possible that some machine learning algorithms can become sensitive. To overcome the class imbalance problem it is possible to oversample the minority class or use a more advanced sampling method like SMOTE (synthetic minority over-sampling technique; Chawla et al., 2002).

Feature selection is primarily focused on removing non-informative or redundant predictors from the model. Many machine learning methods will estimate parameters for every term in the model. Because of this, the presence of non-informative variables can add uncertainty to the predictions and reduce the overall performance of the model. As a first pass, a filter is used to remove features that are highly correlated. Secondly, recursive feature elimination (RFE) is employed to find the set of most informative features (Kuhn and Johnson, 2013).

### Machine Learning Algorithms and Software Used

Classification models were built utilizing the CARET package (short for Classification And REgression Training) version 6.0.84 package in the R programming language (version 3.6.1; R Core Team, 2019).

A long list of available machine learning algorithms is supported by the CARET package. For this analysis, the predictive performances of commonly used machine learning techniques with a binary outcome variable were used (Table 7). Overviews of the various machine learning methods can be found in Kuhn and Johnson (2013) and Tollenaar and van der Heijden (2013).

## Results

We will successively present the predictive validity of the NCRA for general recidivism models with ROC curves and AUCs. We first look at four different feature sets and seven machine learning methods (Table 7) in order to make an overall judgment on the performance of the NCRA. The ROC curves that follow are used as a quantitative assessment of the machine learning methods and the feature sets for which the AUC are summarized into a single AUC number.

**FIGURE 2:**
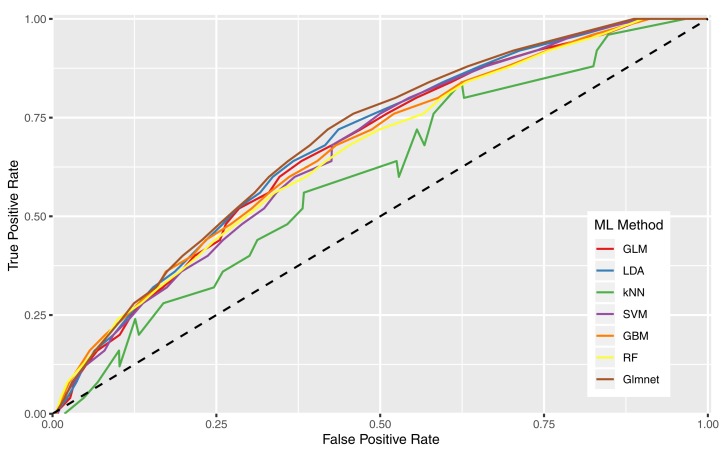
Receiver operating characteristic curves illustrating predictive performance of all machine learning algorithms when looking at the RFE NCRA + Demographics feature set.

Table 8 shows the AUCs for each combination of previously described feature sets and machine learning algorithms. Overall, the Glmnet and LDA algorithms performed similarly well, with the former producing slightly higher AUC across every feature set. ROC curves in Figure 2 show all machine learning methods for the RFE NCRA + Demographics feature set, which corresponds with the reported AUCs in the last row of Table 8.

**TABLE 8:** ROC curve AUCs by feature sets along with machine learning algorithms used.

**Feature sets**	**GLM**	**LDA**	**k-NN**	**SVM**	**GBM**	**RF**	**Glmnet**
Full NCRA	0.60	0.61	0.56	0.64	0.63	0.60	0.64
RFE NCRA	0.64	0.65	0.58	0.66	0.64	0.62	**0.66**
Full NCRA + Demographics	0.65	0.66	0.59	0.65	0.66	0.63	0.69
RFE NCRA + Demographics	0.68	0.69	0.60	0.67	0.67	0.66	**0.70**

The GLM with ridge and lasso regularization (Glmnet) machine learning method performs the best overall for each feature set. On average LDA, GBM, SVM, and GLM performed second highest with very little difference between the methods. The lowest performing machine learning method is k-NN, which is unsurprising given the simplicity of the method. In general, observations on the machine learning methods are in line with the findings of Tollenaar and van der Heijden (2013, 2019).

We next explored whether AUCs improved when we addressed class imbalance by oversampling the minority class. For all machine learning methods (except SVM) using the SMOTE method to correct the imbalance did not improve the predictive validity. At a minority imbalance of 17.3% the imbalance is insufficient to cause problems for most machine learning methods.

**FIGURE 3:**
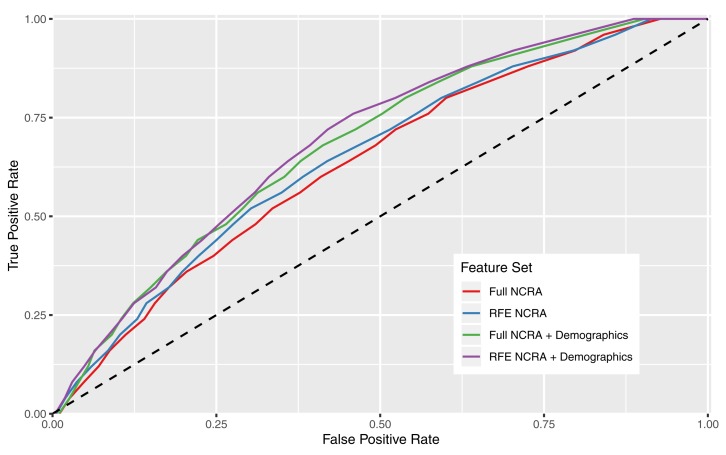
Receiver operating characteristic curves illustrating predictive performance of the Glmnet machine learning method over all feature sets.

Receiver operating characteristic curves in Figure 3 show the performance of the various feature sets for the Glmnet machine learning method, which also corresponds with the reported AUCs in the last column of Table 8. Whether demographics were included or not, feature sets that eliminate features without predictive power (RFE sets) perform better: on average, the AUCs increased by 0.02. The RFE technique is a useful algorithm to identify which individual features are stronger predictors, exclude the poorer performing features, and focus the machine learning algorithms on those reduced sets.

We next found that adding in general information about the participant age, gender, and the current crime category (Table 2) slightly enhanced the predictive performance of the models. This is not surprising, given the established literature on gender in crime, the age–crime curve, and differing recidivism rates for different crime categories. On average, the model produced an AUC 0.04 higher when we included this information; however, the amount of improvement depended on the machine learning method: there was little improvement for SVM, and the most for GLM, LDA, and Glmnet.

**FIGURE 4:**
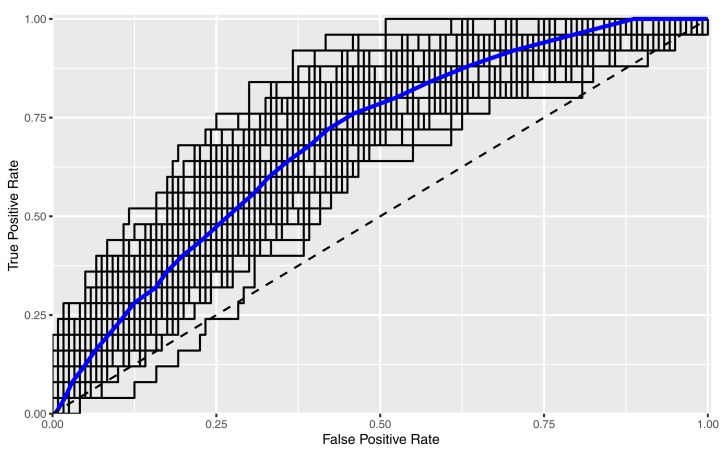
Receiver operating characteristic curves illustrating predictive performance of the Glmnet machine learning method for the RFE NCRA + Demographics feature set.

Receiver operating characteristic curves in Figure 4 show the combination of machine learning algorithm *Glmnet* with the *RFE NCRA* + *Demographics* feature set. The blue line is the average of 100 runs with different splits between training and validation sets, and each individual run is shown by a black line. The amount of variability between individual runs is due to the relatively small sample size for machine learning, and will reduce as the sample size increases. The average ROC produces an AUC of 0.70 which falls between the uppermost bin of the “good” category and “excellent” category for predictive performance indicators (Desmarais and Singh, 2013).

In line with our hypothesis, the feature set of *Full NCRA* with no other information and using a *Glmnet* machine learning algorithm has a relatively competitive AUC that only dipped slightly to 0.66, which is comparable to reported predictive validity in commonly used risk assessments (Table 1), and ranked in the “good” category (Desmarais and Singh, 2013). Because this analysis is based on a small sample size for the purposes of presenting preliminary results, further study is needed to (1) confirm that the AUC increases with more data points (Berk and Bleich, 2013) and (2) allow more time for participants’ possible re-arrests.

Finally, we hope to be able to subset by crime type in the near future, as our data pool expands. As an exploratory step, we split our data into felony and misdemeanor crime level. Despite a reduction in sample size from splitting the data, there appears to be promise that the NCRA will be able to produce predictive scores for general recidivism in either category. Preliminary results show an AUC for felony level crimes at 0.72, a moderate to strong effect with an AUC for misdemeanor level crimes showed an AUC of 0.68. Next steps will include further exploration of felony versus misdemeanor, comparing violent with non-violent crime levels, as well as other detailed level sub-crime types.

### Strengths and Limitations

This study is the first to show that neurocognitive tests optimized as games on a mobile tablet has a predictive accuracy, measured by AUC, comparable to commonly used risk assessments. Further, by using neurocognitive testing – with no racial information – we are able to address important critiques about implicit and explicit racial bias. Further, our approach also avoids the possible confounds of static factors (e.g., using distant criminal history to inform future behavior). Additionally, as the development of the NCRA proceeds, we will adopt an algorithmic equity checklist (Osoba et al., 2019) to minimize any undesirable equity outcomes.

The NCRA is a flexible tool in terms of administration, implementation, and utility. By combining neurocognitive tests with existing actuarial assessment protocols, the benefit of a deeper understanding of deviant decision-making can be factored into sentencing and treatment programs. There is also room for expanding the test into other areas of cognitive testing by adding new tests and conducting additional feature exploitation to carve out the most predictive variables.

We note several data limitations. Our sample size is on the low side for machine learning models; nonetheless, it is high enough to maintain a stable predictive model over multiple runs. As new data are added, we expect the predictive validity will increase. However, no matter how good our test gets in the future, note that no predictive test will ever approach perfection: life and behavior are simply too complex for that.

Another limitation is the crime level distribution of prison-eligible offenders. Felony-level violent offenders and recidivists are represented in the sample; however, participants in this analysis were offenders who committed crimes that were eligible for probation or pre-trial diversion. Despite using two databases to track rearrests both locally and in border counties, the study is limited by siloed jurisdictional databases, which undercount arrest rates for all participants who may have gone on to commit crime in other states.

Also, it is possible that data from this probation sample in Houston may not generalize well to other jurisdictions, and results may differ at key points of the criminal justice pipeline (e.g., pretrial versus pre-sentencing).

## Discussion

The purpose of this study was to analyze the predictive accuracy as measured by AUC, of recidivism in a community probation population using gamified neurocognitive testing. Our results demonstrate that a rapid, gamified, test on a tablet computer can perform as well (or, in many cases better) than the most commonly used risk assessments.

As the use of risk assessments has grown, so has the scrutiny of their efficacy, methods, and purpose, especially in light of equality in machine learning algorithms (Eaglin, 2017; Berk et al., 2018). Following current trends, it is likely that an increasing number of states will mandate risk assessments for defendants and offenders. Recent legislation has been drafted to adopt such policies on a large scale, such as the Pre-trial Integrity and Safety Act. This proposed legislation drew from the implementation of risk assessments in Kentucky to expand such programs in the United States (Harris and Paul, 2017). The current work adds an instrument to the toolbox that can deliver both large-scale social and direct economic impacts.

The NCRA offers a variety of benefits with a predictive validity comparable to widely used risk assessments. This is the first tool to assess the underlying neurocognitive drivers of decision-making in a criminal justice setting. The NCRA has the potential to become a time- and resource-saving option for arraignment assessments. Improvements in predicting re-offense have the potential to translate into a safer society by more effectively modulating sentencing and steering rehabilitative strategies.

Our next steps will be aimed at studying and deploying the NCRA at key points of the criminal justice continuum. It would be beneficial to test individuals at other points of the system, including pre-trial to help determine bail options, probation assessment, jail, and prison intake when rehabilitation programs are assigned. At the end of the pipeline, we’re interested in exploring testing re-entry programs, parole supervision, and explore juvenile justice pipelines. With greater variability in testing timepoints, and also with population, a richer picture can emerge of the trajectory of decision-making.

Outside of the courts, the assessment may help assist in determining beneficial diversion, reentry, or community-based programs for individuals reentering society post conviction, which would call for applying customized thresholds. With a cognitive/behavioral snapshot of an individual, we hope to be better able to address individual need for each person to receive the rehabilitation programs they need to succeed.

To ensure that we do not introduce address racial bias, we did not use any information about race, number of previous arrests, education, or employment in our machine learning models. The best-performing model did include general information such as age, gender, and crime level information. However, we are able to show the small gap between using those factors (0.70 AUC) and using cognitive performance alone (0.66 AUC) showing promise that with a larger sample size, we can drop age, gender, and crime level all together when appropriate (Skeem et al., 2016; Douglas et al., 2017) – yielding a test that only uses neurocognitive measures to predict reoffense with higher AUCs than standard risk assessments.

Previous psychometric evaluation of the NCRA suggested that predictors may exist that would correlate to specific crime subtypes, thereby increasing the predictive performance for a particular type of criminal offender (Ormachea et al., 2017). With increased sample size, we will apply the predictive model to criminal subtypes (e.g., arson, mass shooting) and hope to be able to draw connections between neurocognitive domains and more specific crimes. By examining criminal subtypes and identifying neurocognitive areas of interest, we hope to be able to better understand the drivers of crime, and also offer more effective and targeted rehabilitation direction for specific services.

The NCRA is able to predict reoffense at a level comparable to current risk assessments and has the additional benefits of being intuitive to use, easy to interpret, and uniformly deployable across age, gender, and crime level. Additionally, it holds the potential to give previously unavailable insights into the underlying neurocognitive drivers of decision making of criminal behavior.

## Data Availability Statement

The datasets for this manuscript are not publicly available because the data contain private health information for a protected participant class (prisoners and probationers). Requests to access the datasets should be directed to davideagleman@stanford.edu.

## Ethics Statement

The studies involving human participants were reviewed and approved by the Solutions IRB. The participants provided their written informed consent to participate in this study.

## Author Contributions

GH, SD, and DE contributed to the conception and design of the study. GH, SD, PO, and DE contributed to the assessment invention. DW collected data for the study. GH performed all of the statistical analysis and machine learning. GH, SD, ES, PO, and DE wrote the manuscript.

## Conflict of Interest

The authors declare that the research was conducted in the absence of any commercial or financial relationships that could be construed as a potential conflict of interest.
